# The Effectiveness of Different Interventions to Promote Poison Prevention Behaviours in Households with Children: A Network Meta-Analysis

**DOI:** 10.1371/journal.pone.0121122

**Published:** 2015-04-20

**Authors:** Felix A. Achana, Alex J. Sutton, Denise Kendrick, Persephone Wynn, Ben Young, David R. Jones, Stephanie J. Hubbard, Nicola J. Cooper

**Affiliations:** 1 Clinical Trials Unit, Warwick Medical School, University of Warwick, Coventry, CV4 7AL, United Kingdom; 2 Department of Health Sciences, University of Leicester, Leicester, LE1 7RH, United Kingdom; 3 Division of Primary Care, University of Nottingham, Nottingham, NG7 2RD, United Kingdom; Institute for Health & the Environment, UNITED STATES

## Abstract

**Background:**

There is evidence from 2 previous meta-analyses that interventions to promote poison prevention behaviours are effective in increasing a range of poison prevention practices in households with children. The published meta-analyses compared any intervention against a “usual care or no intervention” which potentially limits the usefulness of the analysis to decision makers. We aim to use network meta-analysis to simultaneously evaluate the effectiveness of different interventions to increase prevalence of safe storage of i) Medicines only, ii) Other household products only, iii) Poisons (both medicines and non-medicines), iv) Poisonous plants; and v) Possession of poison control centre (PCC) telephone number in households with children.

**Methods:**

Data on the effectiveness of poison prevention interventions was extracted from primary studies identified in 2 newly-undertaken systematic reviews. Effect estimates were pooled across studies using a random effects network meta-analysis model.

**Results:**

28 of the 47 primary studies identified were included in the analysis. Compared to *usual care intervention*, the intervention with *education and low cost/free equipment* elements was most effective in promoting safe storage of medicines (odds ratio 2.51, 95% credible interval 1.01 to 6.00) while interventions with *education*, *low cost/free equipment*, *home safety inspection and fitting* components were most effective in promoting safe storage of other household products (2.52, 1.12 to 7.13), safe storage of poisons (11.10, 1.60 to 141.50) and possession of PCC number (38.82, 2.19 to 687.10). No one intervention package was more effective than the others in promoting safe storage of poisonous plants.

**Conclusion:**

The most effective interventions varied by poison prevention practice, but *education alone* was not the most effective intervention for any poison prevention practice. Commissioners and providers of poison prevention interventions should tailor the interventions they commission or provide to the poison prevention practices they wish to promote.

**Highlights:**

- Network meta-analysis is useful for comparing multiple injury-prevention interventions.
- More intensive poison prevention interventions were more effective than *education alone*.
- *Education and low cost/free equipment* was most effective in promoting safe storage of medicines.
- *Education*, *low cost/free equipment*, *home safety inspection and fitting* was most effective in promoting safe storage of household products and poisons.
- *Education*, *low cost/free equipment and home inspection* were most effective in promoting possession of a poison control centre number.
- None of the intervention packages was more effective than the others in promoting safe storage of poisonous plants.

## Introduction

Globally poisonings result in approximately 45,000 deaths[1] and approximately 2.4 million disability adjusted life years (DALYS) lost (GBD 2010) each year in children and young people aged 0–19 years. They are a particular problem in young children with 13% of deaths[1] and 11% of the DALYs lost (GBD 2010) occurring in children aged 0–4 years. Each year, poisonings result in approximately 25,000 emergency department attendances in the UK in 0–4 year olds [2], 63,000 ED attendances in the USA for drug poisoning alone in the 0–5 year olds[3] and more than 1.2 million calls to poison control centres each year following poisoning in the under 5s in the USA[4]. Poisonings among 0–15 year olds have been estimated to cost the NHS more than £2 million each year[5]. In the US despite an estimated saving of $7 to $15 for every $1 spent on poison control centres[6], non-fatal poisonings resulted in $48 million medical costs for hospitalisations and ED attendances in 2005 for the under 5s[7].

There is evidence from 2 previous meta-analyses that interventions to promote poison prevention behaviours are effective in increasing a range of poison prevention behaviours [8,9]. The first meta-analysis in 2001 found a modest effect (statistical significance not reported) of interventions in a clinical setting on safe storage of cleaning products[9]. More recent meta-analyses found home safety education, with or without the provision of safety equipment was effective in increasing safe storage of medicines, safe storage of non-medicinal products and increasing availability of poison control centre numbers[8]. These meta-analyses compared any intervention against a “usual care or no intervention” comparison group. The interventions comprised various combinations of education, home safety inspection, provision of free or low cost safety latches for cabinets, drawers or cupboards and fitting of safety latches. Some of these interventions were aimed only at preventing poisonings, but most aimed to prevent a range of injuries and also included the provision of education and other items of safety equipment. Health care commissioners and housing providers, amongst others, have to make decisions about the “best” intervention for preventing poisoning and this requires comparisons between these multiple interventions. Therefore meta-analyses which have been conducted to date that combine effects across all interventions and compare against usual care or no intervention can only make a limited contribution to these decisions.

Network meta-analysis (NMA) methods[10,11] (also known as mixed treatment comparison[12,13]) extend standard (pair-wise) meta-analysis to allow simultaneous comparison of all evaluated interventions within a single coherent analysis. Therefore all interventions can be compared with one another, including comparisons not evaluated within any of the primary studies. These analyses are increasingly being used in health technology assessment to help decide on the optimal intervention for a particular condition[14]. The objective of this research was to evaluate the effectiveness of different interventions to promote poison prevention behaviours by households with children. We believe this is the first application of NMA in this area of injury prevention.

## Methods

### Study identification

For the NMAs, data on the effectiveness of poison prevention interventions was extracted from primary studies identified in 2 newly-undertaken systematic reviews: a systematic review of reviews [15] and a systematic review of primary studies published since the most comprehensive systematic review [16].

The review included systematic reviews and meta-analyses of experimental study designs (randomised controlled trials (RCTs), non-RCTs and controlled before-and after (CBA) studies) and controlled observational studies (case control and cohort studies), and primary studies of experimental or controlled observational designs published since the most recently published comprehensive systematic review. Studies including children aged 0–19 years and their families that provided interventions to promote poison prevention behaviours were included. Legislative interventions to reduce poisonings were excluded. Interventions to promote possession of ipecac were reported in many papers included in the systematic review, but were excluded from the NMA as use of ipecac is no longer recommended[17,18].

We searched MEDLINE, Embase, CINAHL, ASSIA, PsycINFO and Web of Science from date of inception to January 2012. We searched a range of other electronic sources in January 2013 and hand searched the journal Injury Prevention (March 1995—January 2012) and abstracts from 1st-10th World Conferences on Injury Prevention and Control (1989–2010). Reference lists of included reviews and primary studies were searched for relevant citations. Full-text articles were retrieved regardless of language and translated where necessary. The search terms were adapted for study design and the same sources were searched from 2001—January 2012 to identify primary studies. The search strategy used to search Medline and adapted as necessary for other databases is shown in S1 Searches and the other sources searched are given in S2 Searches.

Titles and abstracts of articles were scanned independently by 2 researchers to identify articles to retrieve in full. Where an article appeared to be eligible based on the title, but an abstract was unavailable, it was retrieved in full. Full articles were independently reviewed by 2 researchers using a standard form listing inclusion criteria. Disagreement between researchers was dealt with by referral to a 3^rd^ member of the research team and consensus-forming discussions.

Data was extracted onto a standard form which recorded data on study design, participants, interventions, outcomes and numerators and denominators in each treatment arm. Data were extracted by 2 researchers independently and compared. Any discrepancies were investigated by referral back to the original article by a senior member of the research team. Authors were asked to supply individual participant data (IPD) or unpublished aggregated data where the published data did not report numerators and or denominators or intra-class correlation coefficients (ICCs) for clustered data. Where studies did not adjust for clustered allocation of intervention, we estimated the effective sample size based on the design effect using published ICCs[19] or ICCs estimated from IPD where the author provided it.

The quality of included primary studies was assessed in terms of the following criteria: allocation concealment, blinding of outcome assessment and completeness of follow up for randomised studies, and blinding of outcome assessment, completeness of follow up and balance of confounders between treatment arms for non-randomized studies. Non-randomised studies were considered to be balanced in terms of confounders if the prevalence of confounders did not differ by more than 10 percent between the treatment arms, and unclear with respect to balance of confounders if the intervention and control groups were matched on various characteristics but no data was provided to judge the adequacy of this matching. The quality of controlled observational studies (case control and cohort studies) was assessed using the Newcastle-Ottawa scale[20].

### Statistical methods

The five poisoning prevention outcomes considered in the NMAs were i) safe storage of medicines (Yes/No), ii) safe storage of other household products (Yes/No), iii) safe storage of poisons (Yes/No), iv) safe storage of poisonous plants (Yes/No), and iv) possession of poison control centre (PCC) telephone number (Yes/No). The safe storage of poisons outcome refers to storage of any potentially toxic substance and includes studies where the reported outcome included both medicines and other household products (i.e. where the outcomes were not reported separately). Safe storage was defined as storing potentially toxic substances (medicinal or non-medicinal) at adult eye level and/or in locked cupboards/drawers/cabinets where they are inaccessible to children [16].

For each of the outcomes, NMA[11] was implemented to enable the comparison of all interventions with one another using all the available data in a connected network of studies; thus allowing comparisons of interventions not directly compared in studies but linked through a connected network of studies (indirect evidence). For example, a comparison of the following 4 interventions; usual care, education, equipment giveaway and home inspection, could be achieved using studies containing the following pair-wise comparisons, usual care *vs*. education, education *vs*. equipment giveaway, equipment giveaway *vs*. home inspection. However, if only studies of usual care *vs*. education and equipment giveaway *vs*. home inspection existed then the network would be *disconnected*; in such cases the analysis would be limited to performing only direct pairwise comparisons. For randomised trials, NMA preserves the within-study randomised treatment comparison of each trial while combining all available comparisons between interventions. Such analyses assume that there is consistency across evidence. For example, if an equipment giveaway arm had been included in the studies of usual care *vs*. education the estimate of education *vs*. equipment giveaway would be consistent across studies (i.e. the underlying estimates are assumed to be identical or exchangeable depending on whether fixed or random effects are assumed) with education *vs*. equipment giveaway.

For these analyses, a standard NMA random effects model with a binary outcome [11,12] was fitted to the data. We obtained pooled estimates of intervention effects for all combinations of pair-wise comparisons from the NMAs and for completeness we also present estimates from the head-to-head evidence for each pair-wise comparison where available. Effectiveness estimates are presented as odds ratios and summarised using forest plots developed by Tan et al[21]. Interventions were ranked based on absolute intervention effects (derived using a underlying rate based on the usual care arms) and the probability that each intervention is best for a particular outcome[11,12] was calculated.

To assess the goodness of fit of the model to the data, the posterior mean residual deviance (defined as the difference between the deviance for the fitted model and the deviance for the saturated model, where the deviance measures the fit of the model to the data points using the likelihood function[22] was calculated. Under the null hypothesis that the model provides an adequate fit to the data, it is expected that the posterior mean residual deviance would have a mean equal to the number of unconstrained data points[23,24].

The between-study standard deviation parameter, τ was used to quantify the heterogeneity of the network (i.e. the variability in treatment effects within pair-wise comparisons above that expected by chance)[25]. The degree of heterogeneity was assessed as reasonable, high or extremely high based on guidelines for interpreting τ on the log-odds ratio scale suggested by Spiegelhalter *et al*. [26]. These state that values of τ from 0.1 to 0.5 may be considered as indicating a reasonable degree of heterogeneity, 0.5 to 1 as high and values above 1 as very extreme heterogeneity. In NMA it is important to assess consistency between the ‘direct’ and ‘indirect’ evidence of the dataset; this was evaluated using a method based on ‘node splitting’[27] which calculates the probability that the mean treatment effect estimates based on the direct evidence (i.e. studies that directly compared the two treatments under consideration) exceeds the mean treatment effect estimates based on the indirect evidence (i.e. the remaining studies in the network under the consistency assumption). A 2-sided p-value was then derived (using the formula 2 x minimum(*prob*, *1-prob*)[27]. Note that only pairs of interventions that are part of a closed loop in the network of interest have both direct and indirect evidence available[13] and therefore can be assessed for consistency.

### Sensitivity analysis

As the quality of included studies varied, analyses for all outcomes except safe storage of poisonous plants were repeated restricted to data obtained from RCTs only. It was not possible to conduct this repeat analysis for safe storage of poisonous plants as only 3 studies provided data for this outcome.

All of the analyses were conducted using a Markov chain Monte Carlo method and fitted in the WinBUGS software[28]. Further technical details of the analysis together with the WinBUGS code are available from the corresponding author. The analysis and subsequent reporting adhere to the PRISMA statement (S1 Prisma Checklist) guidelines[29] and the implied criteria for reporting the results of NMA outlined in Bafeta et al[30].

## Results

**Fig 1:**
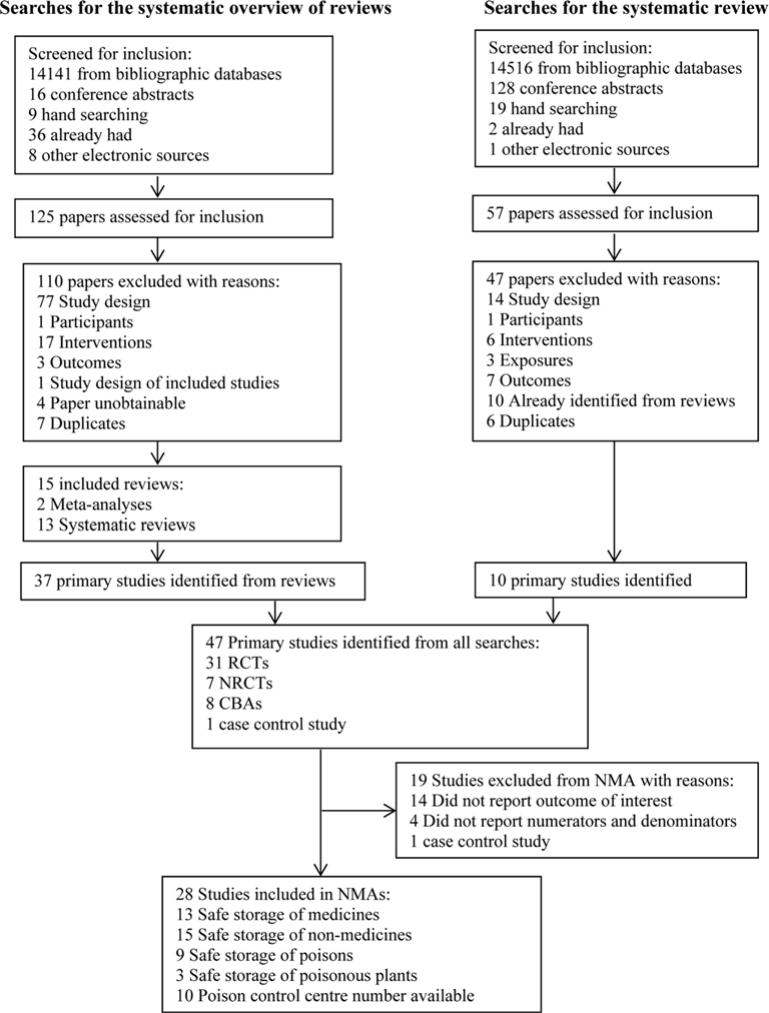
PRISMA flow chart for the systematic overview of reviews and systematic review of primary studies.

The process of selection of studies is shown in Fig 1. One hundred and eighty two papers were assessed for inclusion. This included 125 papers from the search for systematic reviews and 57 from the search for primary studies published since the review we considered to be most comprehensive [31] which was published in 2001. In total 47 primary studies were identified for inclusion in the review, of which 27 were selected for inclusion in at least one of the NMAs (S1 Table).

One study[32] divided patients into a randomised and a quasi-randomised study groups and analysed the two groups separately. This study was therefore counted as two separate studies, thus increasing the total number of studies included in the NMAs to 28. A detailed description of the characteristics of included studies have been published elsewhere [15,16]. A table of studies excluded from the NMA is given in the accompanying supplementary material (S2 Table).

Summary characteristics of the 28 studies included in the NMAs together with their study quality which was observed to be variable across studies are reported in the accompanying supplementary material (S1 Table). Twenty (71%) of the 28 studies were RCTs and 8 (29%) were non-RCTS. Overall, the following 9 intervention packages were evaluated across the 5 networks and no single study compared all interventions directly:

1. Usual care—including usual safety education or no education (UC)
2. Education—more than usual safety education (E)
3. Education + free/low cost equipment (E+FE)
4. Education + free/low cost equipment + home safety inspection (E+FE+HSI)
5. Education + free/ low cost equipment + fitting (E+FE+F)
6. Education + home safety inspection (E+HSI)
7. Education + free/low cost equipment + fitting +home safety inspection (E+FE+HSI+F)
8. Education + home visit as part of Healthy Steps for Young Children program (E+HV) and
9. Free/low cost equipment only (FE).

The free/low cost equipment component of interventions varied between studies [15,16] and included items such as a smoke alarm, batteries, cabinet and window locks, fire guards and stair gates, among others. A detailed list of the equipment reported by each study is presented in are reported in the accompanying supplementary material (S1 Table). Fitting refers to installation of safety equipment by for example a researcher or professional as part of the intervention package [33,34].

### Storage of medicines

**Fig 2:**
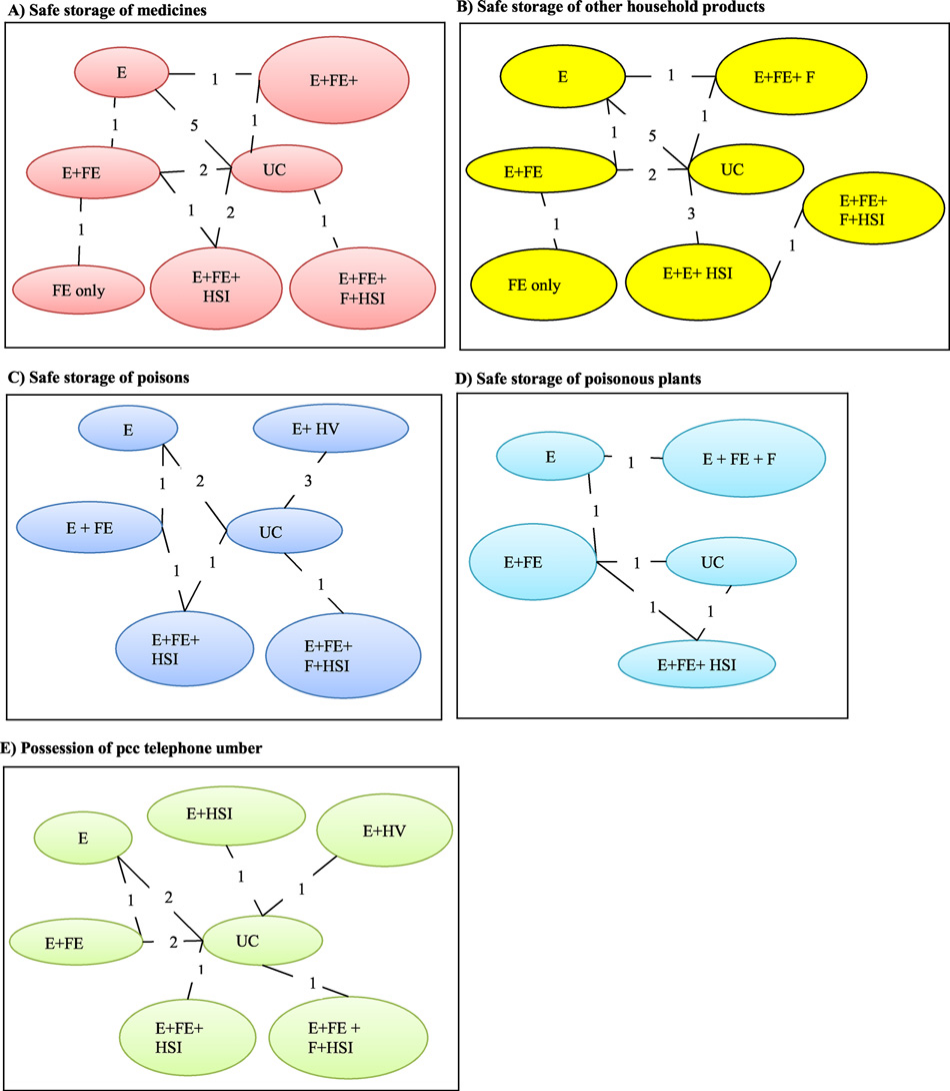
Network Diagrams of interventions to increase safety practices to prevent poisonings in pre-school children in the home. PCC = poison control centre telephone number. Nodes/oval circles represent an intervention (E = education, F = Fitting, FE = low cost/free equipment, HSI = Home safety inspection, HV = Home visit). For example E+FE+HSI = Education + low cost/free equipment + home visit intervention. The lines connecting any two nodes represent the pairwise comparison. The numbers on each line represent the total number of studies and the number of non-RCTs (in brackets) contributing to each pairwise comparison.

Thirteen of the 28 studies compared the effectiveness of 7 interventions to promote safe storage of medicines (Panel A, Fig 2). Eleven (85%) studies were RCTs and 2 (15%) were non-RCTs (S1 Table). One study[35] reported a 100% event rate in both treatment and control arms. This study was excluded from the analysis as it contributed no information on relative effectiveness of the interventions that is of interest in the analysis.

**Fig 3:**
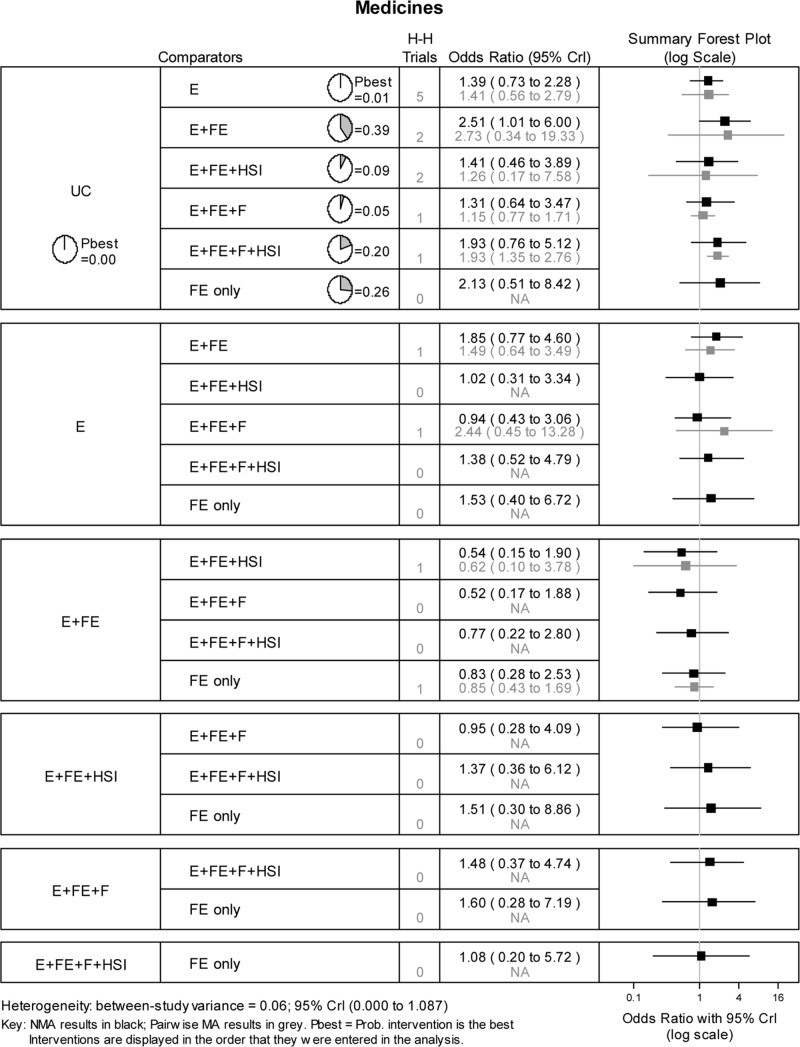
Network meta-analysis results for safe storage of medicines. H-H trials refer to the number of head-to-head trials available for the specified pairwise comparison.

Pooled estimates of 21 possible pairwise comparisons between the 7 interventions, together with the available direct within-trial estimates, are reported in Fig 3. The results show that home safety interventions increase safe storage of medicines with education and low cost/free equipment the most likely to be effective (probability best = 0.39), with an estimated odds ratio compared to usual care of 2.51 (95% CrI: 1.01 to 6.00). When the effect of study design on the NMA results was assessed, by repeating the above analysis using only data from the 11 RCTs, the results were similar, although for this analysis the network was limited to only 6 interventions (i.e. excluding the intervention education, low cost/ free equipment and home safety inspection).

### Storage of other household products

Fifteen studies evaluated 7 interventions for promoting safe storage of other household products (Panel B, Fig 2) of which 11 (73%) studies were RCTs and 4 (27%) were non-RCTs (S1 Table). One study (Dershewitz 1977) reported zero events (i.e. none of the households surveyed safely stored other household products) in the equipment only (9) intervention arm. To facilitate inclusion of this study in the analysis, a continuity correction was applied by adding 0.5 and 1 to the denominator and numerator.

**Fig 4:**
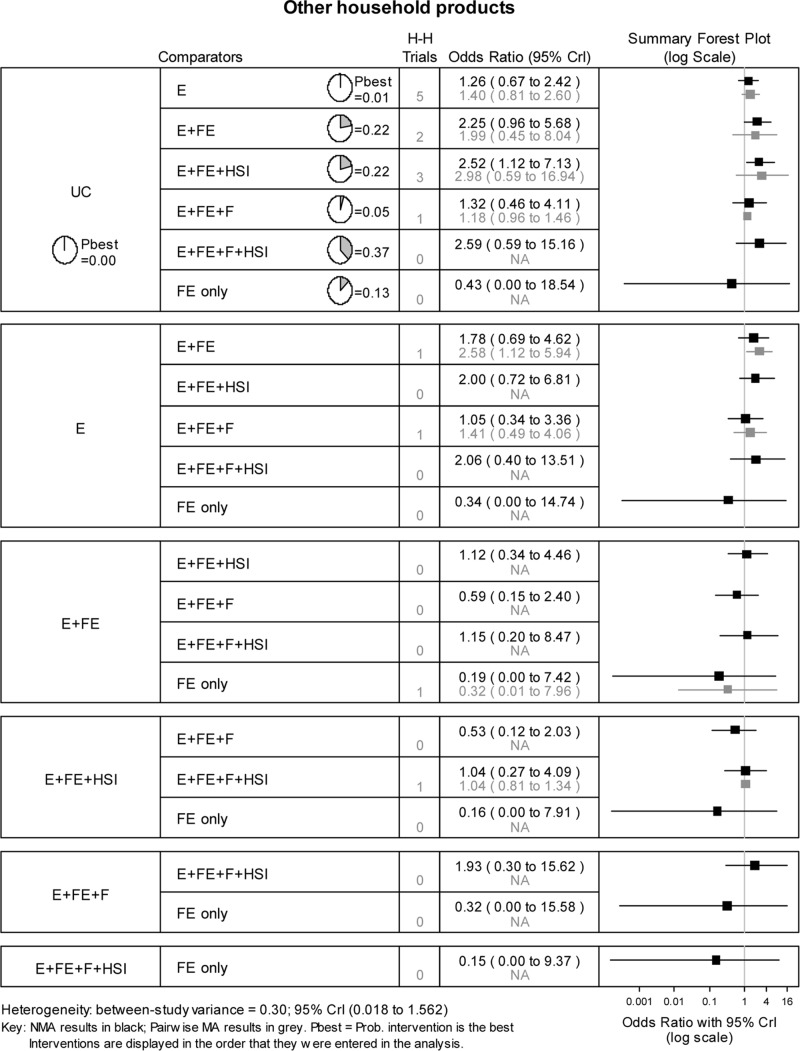
Network meta-analysis results for safe storage of other household products. H-H trials refer to the number of head-to-head trials available for the specified pairwise comparison.

The NMA estimated the 21 possible pairwise comparisons between the 7 interventions trialled across the included studies (Fig 4). The most intensive intervention (education, low cost/free equipment, home safety inspection and fitting) was most likely to be effective (probability best = 0.37), with an estimated odds ratio compared to usual care of 2.59 (95% CrI: 0.59 to 15.16).

The effect of study design on the NMA results was assessed by repeating the above analysis using only data from the 11 RCTs limiting the network to 6 interventions (i.e. excluding education only). The results changed slightly but the most intensive intervention was still most likely to be the most effective (probability best = 0.56) closely followed by the intervention education, low cost/ free equipment and home safety inspection (probability best = 0.44).

### Safe storage of poisons

Nine studies provided data on the effectiveness of 5 interventions to increase safe storage of poisons in households with children (Panel C, Fig 2). Six (67%) studies were RCTs and 3 (33%) were non-RCTs (S1 Table).

**Fig 5:**
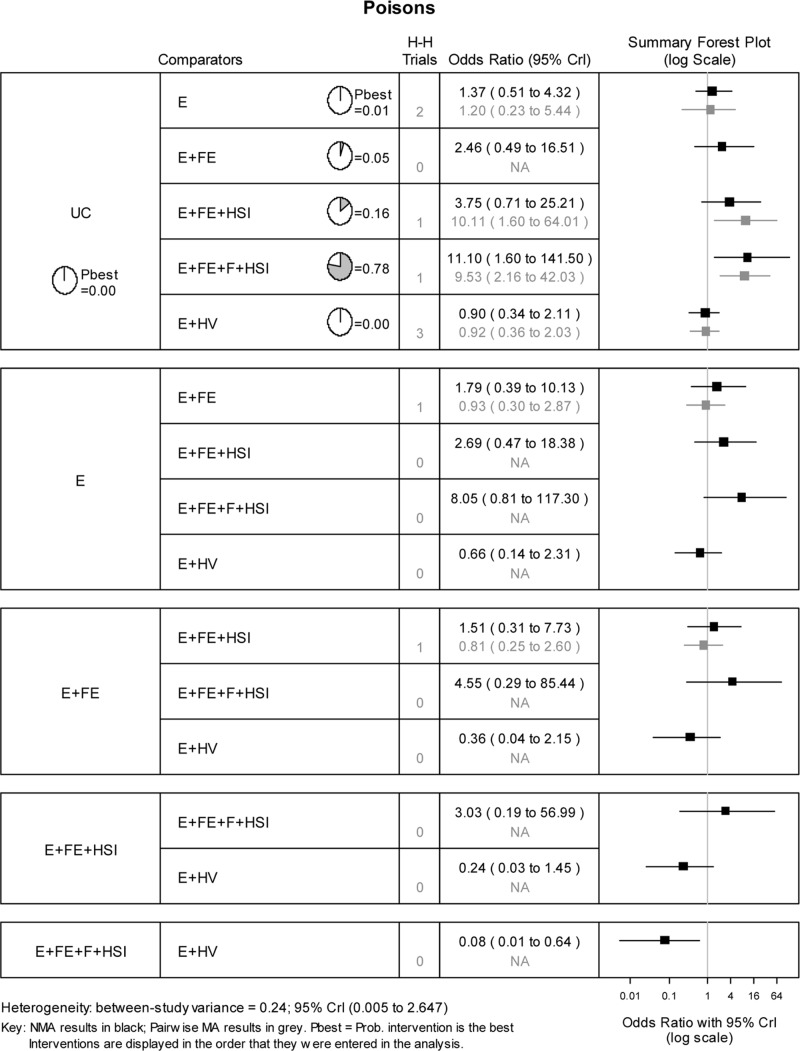
Network meta-analysis results for safe storage of poisons. H-H trials refer to the number of head-to-head trials available for the specified pairwise comparison.

The NMA estimated the 10 possible pairwise comparisons between the 5 interventions trialled across the included studies (Fig 5). There was evidence to suggest that the most intensive intervention (i.e. education, low cost /free equipment, home safety inspection and installation) was most effective in promoting the number of households with storage of poisons compared to usual care intervention (Probability best = 0.78; OR = 11.10, 95% CrI = 1.60 to 141.50).

Repeating the analysis using only data from the 6 RCTs identified both education and low/free equipment (Probability best 0.38), and education, low cost/free equipment, home safety inspection and installation (Probability best 0.36) to be the most effective at promoting the number of households with storage of poisons compared to usual care intervention.

### Safe storage of poisonous plants

**Fig 6:**
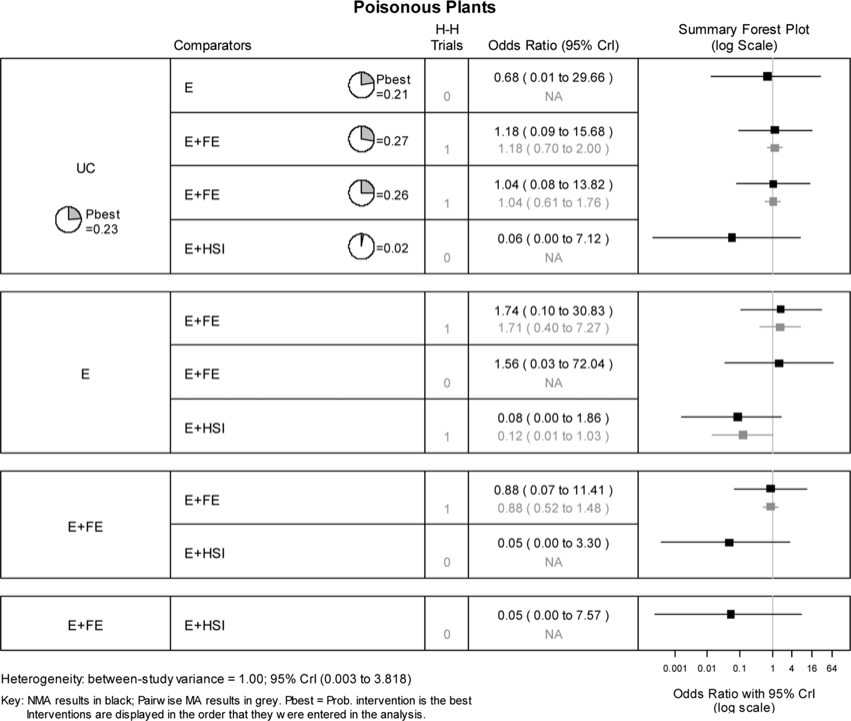
Network meta-analysis results for safe storage of poisonous plants. H-H trials refer to the number of head-to-head trials available for the specified pairwise comparison.

Three RCTs, one of which is the 3-arm study [36] provided data on 5 interventions for storage of poisonous plants (Panel D, Fig 2; S1 Table). The NMA estimated the 10 possible pairwise comparisons between the 5 interventions trialled across the included studies (Fig 6). There was no evidence that any of the intervention was more likely to be effective than the others at promoting safe storage of poisonous plants.

### Possession of a PCC number

**Fig 7:**
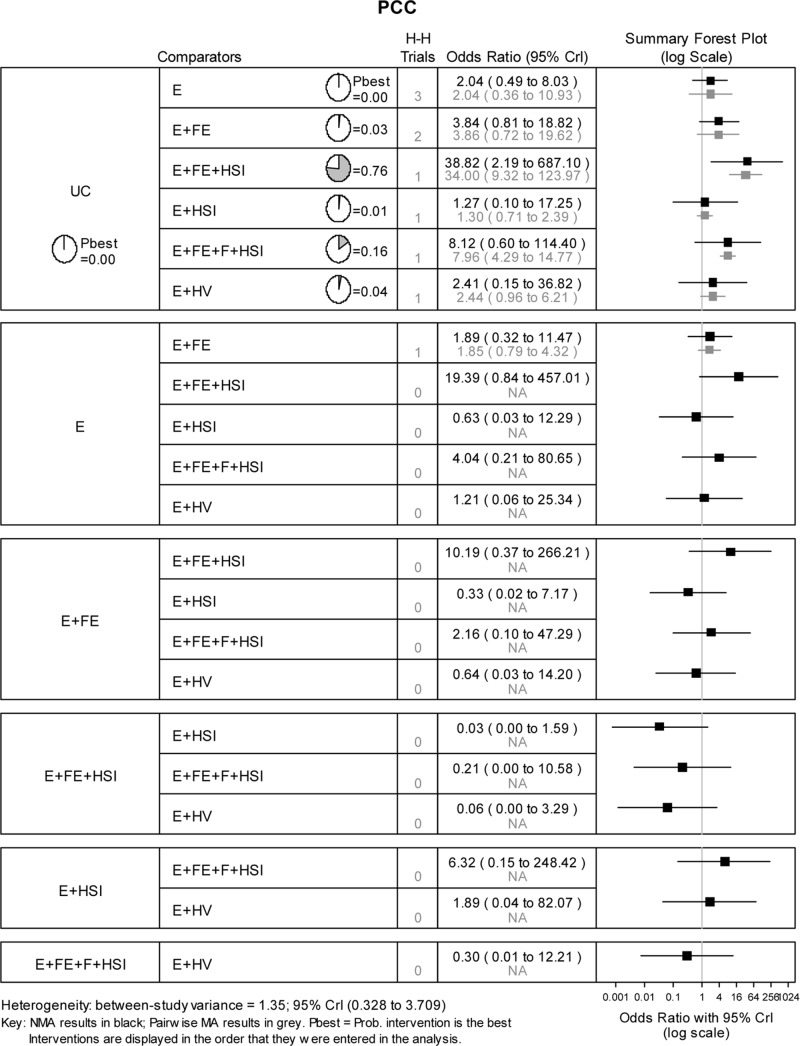
Network meta-analysis results for possession of poison control centre number. H-H trials refer to the number of head-to-head trials available for the specified pairwise comparison.

Ten studies evaluated 7 interventions to promote uptake of PCC number (Panel D, Fig 2). 7 (70%) studies were RCTs and 3 (30%) were non-RCTs (S1 Table). There was evidence that the intervention education, low cost/free equipment and home safety inspection was more effective than usual care in increasing uptake of PCC number (Probability best = 0.76; OR = 39.25, 95% CrI 2.19 to 687.10) (Fig 7). When the effect of study design on the NMA results was assessed by repeating the above analysis using only data from the 7 RCTs based on 5 interventions (i.e. the following 2 interventions were excluded from the network: education and home safety inspection, and education and home visit) the results were very similar.

### Evaluation of models

Overall, the NMA models fitted the data well with the posterior mean residual deviance being close to the number of data points in each network (Table 1). The between study standard deviations for each of the NMA models are reported in Table 1 and indicate moderate between-study heterogeneity for storage of medicines and poisons, high heterogeneity for other household products and extremely high for possession of PCC number. The uncertainty in the estimation of the heterogeneity parameter reflects the relatively low number of studies providing direct evidence for each pairwise comparison. Where both direct and indirect evidence was available, consistency was checked for closed loops (excluding loops formed by multi-arm studies) in the network, using the node-split method. There was no evidence of inconsistency between the direct and indirect evidence in all networks; that is all the p-values were not statistically significant at the 5% significance level (S3 Table).

**Table 1:** Evaluation of model fit.

Outcome	No. of studies	Residual deviance	Posterior median of the between-study standard deviation, τand 95% CrI in brackets
Safe storage of medicines	12	23.5 (cf 24 data points)	0.331 (0.013 to 1.239)
Safe storage of other household products	15	30.9 (cf 30 data points)	0.561 (0.128, 1.270)
Safe storage of poisons	10	21.0 (cf 21 data points)	0.361 (0.029, 1.436)
Safe storage of poisonous plants	3	6.6 (cf 7)	1.00 (0.003 to 3.818
Possession of a PCC number	10	19.5 (cf 20 data points)	1.165 (0.574, 1.926)

## Discussion

### Principal findings

In this NMA, we have been able to compare the different interventions evaluated with one another for promoting poison prevention behaviours by households with children. This analysis has allowed comparisons of strategies not addressed within any of the individual primary studies. The findings showed that more intensive interventions are more effective than education alone for each of the poison prevention practices we evaluated. Education and low cost/free equipment was most effective in promoting safe storage of medicines; education, low cost/free equipment, home safety inspection and fitting was most effective in promoting safe storage of other household products and poisons; and education, low cost/free equipment and home inspection was most effective in promoting possession of a PCC number. There was no evidence that any of the interventions was more effective than the others at promoting safe storage of poisonous plants.

### Strengths and limitations

NMA is a useful synthesis tool for comparing multiple injury prevention interventions which are often complex and multi-faceted, and where the number of studies evaluating the same comparisons is small. NMA enables interventions to be ranked in terms of their effectiveness in promoting safety practices providing results which are more likely to be useful to policymakers, service commissioners and providers when making choices between multiple alternatives than multiple pairwise meta-analyses.

We did not find evidence of inconsistency between direct evidence and indirect evidence in our analyses, although the power to detect inconsistency will have been limited by sparse data, particularly for analyses involving very few studies. The inclusion of non-randomised study designs allowed us to include a greater number of studies in our analysis, but also resulted in the inclusion of studies with greater potential for bias. Eight (29%) of the 28 studies included in our analysis were non-RCTs. Of these, only 2 were assessed as not balanced or unclear in terms of the distribution of confounders between study-arms was unclear is reported (S1 Table). Sensitivity analyses restricting analyses to RCTs produced similar results suggesting our findings were robust to exclusion of non-randomised studies. The quality of studies included in our analyses (assessed in terms of allocation concealment (RCTs only), blinded outcome assessment, balance of confounders (non-RCTs only) and completeness of follow-up) was variable. It was not possible to explore the impact of the individual measures of quality on our results since such an analysis would be extremely limited due to the large number of parameters being estimated in the NMA relative to the number of studies and may even lead to disconnected networks.

Although NMA allows interventions to be classified into more categories than standard pairwise meta-analysis, there is, inevitably, still some “lumping” of interventions within these categories. For example, education may differ in intensity across studies; that is, from a leaflet or brochure distributed by post, to intensive face-to-face classes teaching home safety. Subcategorising the interventions further, to avoid “lumping”, is reliant on detailed information being reported in the primary study publications. However, in the case of poison prevention education, insufficient detail was often reported to enable further sub categorisation.

### Comparisons with existing work

Our findings are consistent with findings from two previous pairwise meta-analyses. DiGuiseppi found interventions promoting “child-proofing” the home delivered in clinical settings had a modest effect (odds ratio 1.8, statistical significance not reported) on safe storage of cleaning products substances[37]. The seconds meta-analysis by the authors of this paper[8], found that education, with or without the provision of safety equipment was effective in increasing safe storage of medicines (OR 1.53, 95% CI 1.27–1.84), safe storage of household products (OR 1.55, 95% CI 1.22–1.96) and, increasing availability of poison control centre numbers (OR 3.30, 95% CI 1.70–6.39). Our findings extend those from the previous meta-analyses by demonstrating which elements of multifaceted interventions are most effective. Furthermore, one of the previous meta-analyses failed to find significant effects of education, with or without the provision of safety equipment on keeping (unspecified) poisons (OR 0.57, 95%CI 0.31–1.07) or plants out of reach (OR 1.18, 0.40–3.48), but we now demonstrate that some poison prevention interventions are effective in promoting these safety practices.

The effect sizes in our NMA for safe storage of medicines, other household products and availability of the poison control centre number are all larger than the effect sizes found in the pairwise meta-analyses previously reported[8,9]. It is likely that, by reducing clinical heterogeneity of interventions, our NMAs may explain some of the statistical heterogeneity in effect sizes found in previous pairwise meta-analyses. Our findings also suggest meta-analyses combining all interventions, (which include less intensive, and as we have shown, less effective interventions) may underestimate the effect of more intensive interventions.

### Implications for practice and research

Our findings suggest that the “best” intervention for increasing a range of poison prevention practices are more intensive interventions. These include, at a minimum, education and providing equipment, but for some poison prevention practices the most effective intervention requires education, equipment provision and fitting and home safety inspection. The most effective intervention varied by poison prevention practice, so commissioners and providers of poison prevention interventions should tailor the interventions they commission or provide to the poison prevention practices they wish to promote. Knowing which interventions are most effective is important, but is only part of the information required to commission or provide poison prevention and cost-effectiveness is an essential part of any decision making process. The effect sizes from this NMA will be used in subsequent decision analyses to determine the most cost effective interventions for increasing poison prevention practices and these analyses will be presented elsewhere. Such an analysis is vital to determine which interventions provide best value for money, as more intensive interventions, which we have shown to be the most effective, will also be the most expensive.

Despite 28 studies being included in at least one NMA, the maximum number included in any NMA was 15 and many comparisons contained only a small number of studies. Further studies are therefore required to increase precision of effect estimates, to increase power to explore effects by study quality and inconsistency between direct and indirect evidence of effectiveness. In addition, a more detailed description of the intervention in future studies, in particular of the content of the educational elements of interventions would be helpful in allowing a finer subcategorisation and exploration of individual educational components. Methods to incorporate individual level data into NMA analyses are now available [38], and these would be useful for exploring whether the effect of interventions vary by characteristics of study population (e.g. deprivation) and the potential impact of interventions on inequalities in prevention practices.

## Conclusions

Network meta-analysis has demonstrated that the most effective interventions varied by poison prevention practice, with more intensive interventions being more effective than education alone for each poison prevention practice. Education and the provision of home safety equipment are important components for all poison prevention practices. Home safety inspections are more important for promoting safe storage of non-medicinal poisons and plants and for possession of PCC numbers. Commissioners and providers of poison prevention interventions should tailor the interventions they commission or provide to the poison prevention practices they wish to promote.

## Supporting Information

S1 Prisma Checklist (DOC)

S1 Searches Medline search strategy for overviews of reviews, systematic reviews and meta-analyses for poison prevention interventions. (DOCX)

S2 Searches Other electronic sources searched. (DOCX)

S1 Table Summary of studies and their data included in the NMA of the interventions to prevent poisonings in children under 5 (Numbers adjusting for clustering in parentheses). (DOCX)

S2 Table Studies excluded from the NMAs. (DOCX)

S3 Table Posterior Means (Mean) and Standard Deviations (Sd) of the log-odds ratios using the full network, direct and indirect evidence on each pairwise comparison. (DOCX)
