# Supplementary material for: The Effectiveness of Different Interventions to Promote Poison Prevention Behaviours in Households with Children: A Network Meta-Analysis
Source: PLoS One. 2015 Apr 20;10(4):e0121122. doi: 10.1371/journal.pone.0121122 (PMC4404249; doi:10.1371/journal.pone.0121122)
Supplement: S1 Searches — (DOCX) [file pone.0121122.s002.docx]

**S1 Search strategy: Medline search strategy for overviews of reviews, systematic reviews and meta-analyses for poison prevention interventions.**

1. review.m_titl.

2. systematic.m_titl.

3. meta-analysis.m_titl.

4. review.pt.

5. meta-analysis.pt.

6. 1 or 2 or 3 or 4 or 5

7. limit 6 to humans

8. exp child/

9. exp infant/

10. exp adolescent/

11. exp minors/

12. (child$ or adolesc$ or infan$ or young$ or toddl$ or bab$).tw.

13. or/8-12

14. exp "early intervention (education)"/

15. exp education/

16. exp public health/ed

17. exp parenting/

18. exp counseling/

19. (educat$ or train$ or teach$ or parent$ or counsel$ or supervis$).tw.

20. exp accident prevention/ or injury prevention.tw.

21. exp safety/

22. exp safety management/

23. safety practice$.tw.

24. safety equipment.tw. or exp equipment safety/

25. exp infant equipment/

26. exp protective devices/ or (protect$ adj3 device$).tw.

27. exp "interior design and furnishings"/

28. exp consumer product safety/

29. exp drug storage/

30. ((medicine$ or drug$) adj3 storage).tw.

31. exp hazardous substances/ae, po or (hazardous adj3 substance$ adj3 storage).tw.

32. exp household products/ae, po or (household adj3 product$ adj3 storage).tw.

33. (((child adj3 resistant) or childproof) adj3 (closure$ or cap$ or container$)).tw.

34. ((cupboard$ or cabinet$ or drawer$ or box$) adj3 ($lock$ or latch$)).tw.

35. (medicine$ or cosmetics or ((clean$ or beauty or make-up or household or hazardous or industrial) adj3 (supplies or products or materials))).tw.

36. ((toiletries or vitamin$ or cigarette$) adj3 (storage or cupboard$ or cabinet$ or drawer$ or box$ or reach or label$)).tw.

37. ((toxi$ or pollutant$ or gas$) adj3 prevent$).tw.

38. ((toxic or poison$) adj3 plant$ adj3 prevent$).tw.

39. exp ipecac/

40. (poison$ adj3 (control or sticker$ or telephone or number or emergenc$)).tw.

41. or/14-40

42. exp accidents/ or exp accidents, home/

43. exp poisoning/

44. exp "wounds and injuries"/

45. (accident$ or poison$ or injur$ or ingest$ or swallow$ or inhal$).tw.

46. or/42-45

47. 7 and 13 and 41 and 46

The above search was adapted to find primary studies published since the most comprehensive review, substituting the terms below for study design:

1. **for experimental study designs:**

1 randomi?ed controlled trial.pt.

2 randomi?ed controlled trials.sh.

3 randomi?ed controlled trial$.mp. or Randomi?ed Controlled Trials/

4 random allocation.sh.

5 double blind method.sh.

6 single blind method.sh.

7 Random Allocation/

8 1 or 2 or 3 or 4 or 5 or 6 or 7

9 Clinical Trials/ or Placebos/

10 CONTROLLED CLINICAL TRIAL.pt.

11 comparative stud$.mp.

12 intervention stud$.mp.

13 control group$.mp. or Control Groups/

14 placebo$.mp. or PLACEBOS/

15 evaluation stud$.mp.

16 9 or 10 or 11 or 12 or 13 or 14 or 15

(b) **for case control and cohort studies:**

1. exp Case-Control Studies/
2. exp Cohort Studies/
3. 1 or 2
