# Supplementary material for: The Effectiveness of Different Interventions to Promote Poison Prevention Behaviours in Households with Children: A Network Meta-Analysis
Source: PLoS One. 2015 Apr 20;10(4):e0121122. doi: 10.1371/journal.pone.0121122 (PMC4404249; doi:10.1371/journal.pone.0121122)
Supplement: S2 Searches — (DOCX) [file pone.0121122.s003.docx]

**S2 Search strategy: Other electronic sources searched.**

Cochrane database of systematic reviews

Database of Abstracts of Reviews of Effects

NHS Economic Evaluation Database and the Health Technology Assessment Database

Injury Prevention Research Centers at the Centers for Disease Control (USA)

National Institute for Health and Clinical Excellence (NICE) (UK)

Children’s Safety Network (USA)

International Society for Child and Adolescent Injury Prevention (International)

Child Accident Prevention Trust (UK)

Royal Society for the Prevention of Accidents (UK)

Injury Control Resource Information Network (USA)

National Injury Surveillance Unit (Australia)

SafetyLit (USA)

The National Research Register (UK) (up to September 2007)

UKCRN Clinical Research Portfolio
