# Supplementary material for: The Effectiveness of Different Interventions to Promote Poison Prevention Behaviours in Households with Children: A Network Meta-Analysis
Source: PLoS One. 2015 Apr 20;10(4):e0121122. doi: 10.1371/journal.pone.0121122 (PMC4404249; doi:10.1371/journal.pone.0121122)
Supplement: S1 Table — (DOCX) [file pone.0121122.s004.docx]

**S4 Table: Summary of studies and their data included in the NMA of the interventions to prevent poisonings in children under 5 (Numbers adjusting for clustering in parentheses).**

| **Comparison** | **Study** | **Study quality** | **List of free and or low cost equipment provided as part of intervention scheme** | **Safe storage of medicines** | **Safe storage of other household products** | **Safe storage of poisons** | **Safe storage of poisonous plants** | **Possession of pcc^2^ telephone number** |
| --- | --- | --- | --- | --- | --- | --- | --- | --- |
| Usual care (1) vs.  Education (2) | Kelly (1987)^a^, RCT, USA | A=U,B=Y,F=N | Not applicable | 54/54  55/55 | 43/54  49/55 |  |  |  |
|  | Nansel (2002)^b^, RCT, USA | A=Y,B=U,F=Y | Not applicable | 83/89  79/85 | 65/89  66/85 |  |  | 59/89  63/85 |
|  | Kelly (2003)^c^, Cluster-RCT, USA | A=U,B=Y,F=Y | Not applicable |  |  |  |  | 45.56/136.68^c^  112.95/137.63^c^ |
|  | McDonald (2005), RCT, USA | A=Y,B=U,F=N | Not applicable | 6/60  4/57 | 3/57  6/61 |  |  |  |
|  | Gielen (2007), RCT, USA | A=Y,B=N,F=Y | Not applicable | 178/271  188/249 | 44/62  57/73 | 222/333  245/322 |  |  |
|  | Nansel (2008), Non-RCT, USA | A=U,B=N,F=N | Not applicable | 72/74  140/144 | 59/73  117/144 |  |  | 50/59  90/119 |
|  | Reich (2011)^c^, RCT, USA |  | Not applicable |  |  | Log-OR(SE) = -0.192(0.2863) ^d^ |  |  |
| Equipment only (1) vs.  Education + Equipment (3) | Woolf (1987), Cluster-RCT, USA | A=U,B=Y,F=N | Sticker with poison control centre telephone number, bottle of ipecac |  |  |  |  | 29/143  47/119 |
|  | Woolf (1992), Cluster-RCT, USA | A=U,B=Y,F=N | Kitchen cabinet locks, a coupon for purchase of syrup of ipecac, and two telephone stickers with the telephone number of the poison centre. |  | 60/151  89/150 |  |  | 59/151  117/150 |
|  | Clamp (1998), RCT, UK | A=U,B=N,F=Y | Smoke alarm, 2 window locks, 3 cupboard locks, 6 socket covers and a door slam device. | 68/82  79/83 | 49/82  59/83 |  |  |  |
| Usual care (1) vs.  Education + Equipment (3) vs.  Education + Equipment + Home Safety inspection (4) | Babul (2007), RCT, Canada | A=Y,B=N,F=N | Smoke alarm, a coupon for 50% savings on a safety gate, corner cushions, cabinet locks, blind cord windups, water temperature card, doorstoppers, electrical outlet covers and a poison control sticker. | 147/149  171/173  160/163 |  |  | 112/147  136/172  123/160 |  |
| Usual care (1) vs.  Education + Equipment + Home Safety inspection (4) | Kendrick (1999), Cluster non-RCT, UK | B=N,F=N,C=Y | Stair gates, fireguards, cupboard locks and smoke alarms. |  | 317/367  322/363 |  |  |  |
|  | Sangvai (2007), RCT, USA | A=Y,B=Y,F=N | Smoke detectors, gun locks, cabinet locks and water temperature cards. |  |  | 3/10  13/16 |  |  |
|  | Swart (2008), Non RCT, South Africa | A=U,B=Y,F=Y | Child-proof locks and paraffin container safety caps. | 70.26/79.58^c^  74.07/80^c^ | 46.86/57.96^c^  50.87/58.27^c^ |  |  |  |
|  | Hendrickson (2002), USA, RCT | A=N,B=N,F=Y | Full publication or report not available to extract information on equipment provision. |  | 14/40  34/38 |  |  | 8/40  34/38 |
| Usual care (1) vs.  Education + Equipment (3) | Watson (2005), Cluster-RCT, UK | A=Y,B=N,F=Y | Stair gates, fire guards, smoke alarms, cupboard locks and window locks. | 683/738  712/762 | 327/669  368/693 |  |  |  |
| Usual care (1) vs.  Education + Home Safety inspection (6) | Petridou (1997), Cluster non-RCT, Greece | B=N,F=Y,C=Y |  |  |  |  |  | 67.26/100.12^c^  71.08/97.83^c^ |
| Usual care (1) vs.  Education + Equipment + Home Safety inspection + Installation (7) | Schwarz (1993), Cluster non-RCT, USA | B=N,F=N,C=Y | Smoke detectors, batteries, bathwater thermometer, nightlight, a bottle of syrup of ipecac, a sticker for the telephone with emergency telephone numbers. | 88.42/248.37^c^  128.16/248.37^c^ |  |  |  |  |
|  | Phelan (2011), RCT, USA | A=Y,B=N,F=Y | Full publication or report not available to extract information on equipment provision. |  |  | 17/149  2/150 |  | 16/138  71/139 |
| Usual care (1) vs.  Education + Home visit (8) | Minkovitz (2003a)^e^, RCT, USA | A=Y,B=N,F=Y | Not applicable |  |  | 463/761  523/832 |  |  |
|  | Minkovitz (2003b)^e^, Cluster non-RCT, USA | B=N,F=Y,C=Y | Not applicable |  |  | 596/955  754/1189 |  |  |
|  | Johnston (2006), non-RCT, USA | B=N,F=Y,C=Y | Not applicable |  |  | 155/232  71/91 |  | 82/91  222/232 |
| Education (2) vs.  Education + Equipment (3) | Posner (2004), RCT, USA | A=Y,B=Y,F=N | Not applicable | 14/47  19/49 | 22/47  34/49 |  | 9/16  11/16 | 27/47  35/49 |
|  | Bulzachelli (2009), Non-RCT, USA | A=U,B=N,F=N | Not applicable |  |  | 5/49  10/105 |  |  |
| Education (2) vs.  Education + Equipment (5) | Sznajder (2003), RCT, France | A=Y,B=N,F=Y | Cupboard and drawer latches, door handle covers, table protection corners, electric outlet covers, a non-skid bathtub mat, a smoke detector, and a phone sticker with the number of the poison control centre. | 44/49  43/45 | 32/41  40/48 |  | 48/49  41/48 |  |
| Education+ equipment (3) vs.  Education + Equipment + Home Safety inspection (4) | Gielen (2002)^c^, Cluster-RCT, USA | A=U,B=U,F=N | Safety products (eg, ipecac syrup, cabinet latches, safety gates, smoke alarms, batteries, and hotwater thermometers) are sold at 10% to 15% below retail cost in a homelike environment where their use can be demonstrated. |  |  | 6.87/56.93^c^  5.89/58.89^c^ |  |  |
| Education+ equipment (3) vs.  Equipment only (9) | Dershewitz (1977), RCT, USA, | A=U,B=Y,F=N | Electric outlet covers and three kindergards, which are easily installed plastic locking devices intended to prevent children from getting into cabinets. | 22/102  20/104 | 1/101  0/104 |  |  |  |
| Education + Equipment + home Safety inspection (4) vs.  Education + equipment + home safety inspection + Fitting (7) | King (2001), RCT, USA | A=Y,B=Y,F=Y | Coupons from a national retail store for a $10 discount per item (to a maximum of $50) when purchasing recommended safety devices. |  | 261/469  273/482 |  |  |  |

1. Abbreviations: A = adequate allocation concealment; B = blinded outcome assessment; C, prevalence of confounders does not differ by more than 10% between treatment arms; CBA, controlled before-and-after study; F = at least 80% participants followed up in each arm; NMA, network meta-analysis; RCT, randomized clinical trial; U = unclear; Y= yes.
2. ^1^Figures are number of events/total number households in the intervention with lowest code followed by the intervention with the highest code
3. ^2^PCC = Poison control centre
4. ^a^ Study was excluded from analysis for safe storage of medicines because both treatment and control arms reported 100% event rate.
5. ^b^ Two intervention arms were combined (tailored advice and tailored advice + care provider feedback)
6. ^c^ Figures adjusted for the effect of clustering using ICC and method reported Kendrick et al (2012)
7. ^d^ Combined from two log-odds ratios for Education book vs. No Book (OR=0.80, SE=0.41) and Education Book vs. Non-Education Book (OR=0.85, SE=0.40) reported in Reich et al. (2011)
8. ^e^ Minkovitz (2003) included as two separate studies (reason given in the results section)
