# Supplementary material for: The Effectiveness of Different Interventions to Promote Poison Prevention Behaviours in Households with Children: A Network Meta-Analysis
Source: PLoS One. 2015 Apr 20;10(4):e0121122. doi: 10.1371/journal.pone.0121122 (PMC4404249; doi:10.1371/journal.pone.0121122)
Supplement: S2 Table — (DOCX) [file pone.0121122.s005.docx]

**S5 Table: Study Excluded From the NMAs.**

| **First author, year of publication** | **Title** | **Reason for exclusion (n/N = number of households with outcome/Total number of household surveyed in the treatment arm)** |
| --- | --- | --- |
| Baudier, 1988 | A cooperative program for the prevention of domestic accidents in children at the department of the doubs. ii. Educative aspects and evaluation | Outcomes - safe storage of poisons - n/N not reported by treatment arm |
| Campbell, 2001 | Possession of fire extinguisher Practised fire escape plan | Outcomes - safe storage of meds & cleaning products - n/N not reported by treatment arm |
| Colver, 1982 | Promoting children's home safety | Outcomes - safety changes made to home, including poison prevention behaviours, n/N not reported by treatment arm |
| Fergusson, 1982 | A Controlled Field Trial of a Poisoning Prevention Method | Outcomes - poisonings, plus mean number poisons in reach, poisoning hazards score, n/N not reported by treatment arm |
| Garcia, 1996 | Safety fairs: evaluation of a school-based injury prevention program | Outcomes - poison safety - n/N not reported by treatment arm |
| Guyer, 1989 | Prevention of Childhood Injuries: Evaluation of the  Statewide Childhood Injury Prevention Program (SCIPP) | Outcomes - poisonings, poison preventive behaviour score, n/N not reported by treatment arm |
| Johntson, 2000 | A preschool program for safety and injury  prevention delivered by home visitors | Outcomes - ipecac, removed poisons from home, disposed of unwanted meds |
| Kendrick, 2007 | ''Risk Watch'': Cluster randomised controlled trial evaluating an injury prevention program | Outcomes - child never gets meds without asking adult, relevant outcome information not reported |
| Lacoutre, 1978 | Evaluation of a Community-Based Poison Education Program | Outcomes – ipecac excluded from the analysis |
| LeBailly, 1990 | The children's safety research project. | Outcomes - ipecac excluded from the analysis |
| LeBlanc, 2006 | Home safety measures and the risk of unintentional injury  among young children: a multicentre case–control study | Study design - case control |
| Odendaal, 2008 | The impact of a home visitation programme on household hazards associated with unintentional childhood injuries: A randomised controlled trial | Outcomes - poisoning score - n/N not reported for intervention and control arms |
| Paul, 1994 | Preventing accidental injury to young children in the home using volunteers | Outcomes - lockable cabinets, possession of ipecac - n/N not reported by treatment arm |
| Scherz, 1968 | Childhood Poisonings from Medications: Three Prevention Programs That Worked | Outcomes - aspirin poisonings, relevant outcome information not reported |
| Steele (a), 1985 | Symposium on injuries and injury prevention: Poisons | Outcomes - poisonings, PCC utilisation rates, relevant outcome information not reported |
| Steele (b), 1985 | Symposium on injuries and injury prevention: Poisons | Outcomes - poisonings, PCC utilisation rates, relevant outcome information not reported |
