# Supplementary material for: The Effectiveness of Different Interventions to Promote Poison Prevention Behaviours in Households with Children: A Network Meta-Analysis
Source: PLoS One. 2015 Apr 20;10(4):e0121122. doi: 10.1371/journal.pone.0121122 (PMC4404249; doi:10.1371/journal.pone.0121122)
Supplement: S3 Table — (DOCX) [file pone.0121122.s006.docx]

| Pair-wise contrast | Combined evidence from NMA model | Direct evidence | Indirect evidence | Inconsistency estimate^†^ | p-value* |
| --- | --- | --- | --- | --- | --- |
| **Safe storage of medicines** |  |  |  |  |  |
| Usual care (1) vs. Education (2) | 0.40 (0.30) | 0.50 ( 0.34) | 0.79 (0.86) | 0.89 ( 0.97) | 0.306 |
| Usual care (1) vs. Education + Free/low cost Equipment (3) | 0.74 (0.61) | 0.48 (1.23) | 0.38 (0.36) | -0.31 (1.46) | 0.820 |
| Usual care (1) vs. Education + Equipment + Fitting (5) | 0.35 (0.49) | 0.14 (0.56) | 1.55 (1.15) | -1.41 (1.27) | 0.232 |
| Education (2) vs. Education + Free/low cost Equipment (3) | 0.34 (0.58) | 0.41 (0.79) | 0.12 (1.20) | 0.29 (1.44) | 0.778 |
| Education (2) vs. Education + Equipment + Fitting (5) | -0.05 (0.54) | 1.24 (1.09) | -0.32 (0.68) | 1.56 (1.27) | 0.189 |
| **Safe storage of non-medicines** |  |  |  |  |  |
| Usual care (1) vs. Education (2) | 0.24 (0.32) | 0.36 (0.38) | -0.24 (0.73) | 0.61 (0.83) | 0.408 |
| Usual care (1) vs. Education + Free/low cost Equipment (3) | 0.82 (0.43) | 0.68 (0.54) | 1.29 (0.91) | -0.61 (1.05) | 0.499 |
| Usual care (1) vs. Education + Equipment + Fitting (5) | 0.30 (0.54) | 0.17 (0.76) | 0.60 (1.01) | -0.43 (1.27) | 0.698 |
| Education (2) vs. Education + Equipment (3) | 0.58 (0.48) | 0.96 (0.85) | 0.34 (0.67) | 0.61 (1.07) | 0.508 |
| Education (2) vs. Education + Equipment + Fitting (5) | 0.07 (0.56) | 0.31 (0.93) | -0.12 (0.83) | 0.43 (1.25) | 0.690 |
| **Safe storage of poisons** |  |  |  |  |  |
| Usual care (1) vs. Education (2) | 0.34 (0.52) | 0.20 (0.46) | 2.72 (1.61) | -2.52(1.68) | 0.118 |
| Usual care (1) vs. Education + Equipment + Home safety inspection (4) | 1.36 (0.93) | 2.39 (1.22) | -0.07(1.32) | 2.46 (1.77) | 0.107 |
| Education (2) vs. Education + Free/low cost Equipment (3) | 0.60 (0.84) | -0.01 (0.88) | 2.61(1.51) | -2.62 (1.76) | 0.124 |
| Education + Equipment (3) vs. Education + Equipment + Home safety inspection (4) | 0.42 (0.82) | -0.28 ( 0.82) | 2.44(1.50) | -2.74 (1.71) | 0.083 |
| **Possession of a PCC number** |  |  |  |  |  |
| Usual care (1) vs. Education (2) | 0.71 (0.68) | 0.70 (0.81) | 0.72 (1.72) | -0.02 (1.89) | 0.989 |
| Usual care (1) vs. Education + Equipment (3) | 1.33 (0.78) | 1.34 (0.97) | 0.69 (0.81) | 0.04 (1.90) | 0.986 |
| Education (2) vs. Education + Equipment (3) | 0.63 (0.879) | 0.63 (1.43) | 0.63 (1.26) | 0.00 (1.91) | 0.992 |
| †inconsistency estimate = direct estimate – indirect estimate of the treatment effect (log-OR)  *p-value = $2\times\left( probability of direct estimate > indirect estimate \right)$ which gives the 2-sided probabilities that the direct and indirect evidence are different | | | | | |

**S6 Table: Posterior Means (Mean) and Standard Deviations (Sd) of the log-odds ratios using the full network, direct and indirect evidence on each pairwise comparison.**
